# Mental health and life-course shocks in a low-income country: Evidence from Malawi

**DOI:** 10.1016/j.ssmph.2022.101098

**Published:** 2022-04-26

**Authors:** Ally Scheve, Chiwoza Bandawe, Hans-Peter Kohler, Iliana V. Kohler

**Affiliations:** a Swarthmore College, USA; b Kamuzu University of Health Sciences (KUHeS), Malawi; c Population Aging Research Center (PARC), University of Pennsylvania, USA; d Population Studies Center (PSC), University of Pennsylvania, USA

**Keywords:** Mental health, Depression, Anxiety, Economic shocks, Poverty, Malawi, Sub- saharan Africa, MLSFH, Malawi Longitudinal Study of Families and Health, MLSFH-MAC, Mature Adults Cohort of the Malawi Longitudinal Study of Families and Health, PHQ-9, Patient Health Questionnaire-9, GAD-7, General Anxiety Disorder-7, SSA, Sub-Saharan Africa, LIC, Low-income country, WHO, World Health Organization, LMICs, Low- and middle income countries

## Abstract

Economic insecurity has been widely hypothesized to be an important determinant of mental health, but this relationship has not been well-documented in low-income countries. Using data from the Mature Adults Cohort of the Malawi Longitudinal Study of Families and Health (MLSFH-MAC), we investigate the association of negative economic shocks with mental health outcomes such as depression and anxiety among adults aged 45+ years living in a low-income country. Using fixed effects estimates that control for time-invariant unobserved individual heterogeneity, we find that increased economic instability caused by events such as death of a family member, yield loss, or income loss is positively associated with worse mental health outcomes as measured by the PHQ-9 and GAD-7 instruments. Our results suggest that costly economic events are a key component to worsening mental health in settings characterized by pervasive poverty and underscore the importance of mental health as a public health and development target.

## Highlights

• Economic shocks are associated with worsening mental health within resource limited populations.
• Death of a family member, loss of agriculture yield, and income loss were particularly detrimental to individual mental health.
• Positive economic shocks such as fertilizer subsidies, were not significantly correlated with changes in mental health.
• Fixed effects analysis control for time invariant unobservables and provide more cred-ible causal estimates.

## 1 Introduction

Depression and anxiety are two important dimensions of mental health that have a significant and growing contribution to the global burden of disease (Collins et al., 2013; Susser & Patel, 2014; Vigo et al., 2016). In resource-poor contexts, depression and anxiety have been widely recognized as having important implications for individual productivity, individual and family-level well-being, and overall economic development (Canavan et al., 2013; Kohler et al., 2017; Lund et al., 2018). While there has been a more concerted effort in recent years by researchers and international organizations such as the World Health Organization (WHO) to understand the determinants of mental illnesses such as depression and anxiety, these mental health issues still are poorly documented and understood, especially in Sub-Saharan Africa (SSA).

One potentially important determinant of depression and anxiety is economic insecurity. This relationship has been explored in high-income countries, such as after the Great Recession. For example, in Austria job loss due to plant closures was correlated with higher subsequent antidepressant use and more mental health related hospitalizations (Kuhn et al., 2009). Similarly, in Greece a cross-sectional study found that the rate of major depressive episodes significantly increased from the beginning of 2008–2009 after the meltdown of the Greek economy (Madianos et al., 2011). Individuals in the United States who reported falling behind on their mortgage payments were found more likely to develop depressive symptoms (Alley et al., 2011). More recently, economic insecurity due to the COVID-19 pandemic has been linked to worsening mental health in the United States and European countries (Kämpfen et al., 2020; Witteveen & Velthorst, 2020). Furthermore, Kämpfen et al., 2020 found that non-white respondents who are more socioeconomically disadvantaged did not experience worsening mental health from fear of contracting COVID-19 but were particularly concerned about the economic consequences of the pandemic. This suggests that those already struggling economically in high-income countries experience pronounced mental health deterioration from negative economic shocks.

While the relationship between negative economic shocks and mental health outcomes has not been explored extensively in low- and middle-income countries (LMIC), there are a number of studies in this setting evaluating the impact of positive economic shocks on mental health via anti-poverty or cash-transfer programs. (Kilburn et al., 2016; Angeles et al., 2019; Ohrnberger et al., 2020a,b). For example, Kenyan households given cash transfers of 400–1500 USD reported increased consumption and happiness and a reduction in depression and stress (Haushofer & Shapiro, 2016). These studies provide compelling associations between positive economic shocks and mental health. However, these experiments do not provide evidence for how negative economic shocks, that are frequently occurring in SSA, impact mental health leading to two questions. First, at extreme levels of poverty will additional negative shocks further increase levels of depression and anxiety? Second, will differing economic events compared to those in high-income countries such as drought or death of an important family member also cause worsening mental health? A study conducted in rural Indonesia is the closest to getting at these questions in a low- and middle income (LMIC) context. Using longitudinal data from the Indonesia Family Life Survey, Christian et al. (2019) explored the impacts of rain fall on mental health and found that lack of rain fall was correlated with higher depression scores. However, it is worth noting that Indonesia was recently re-categorized as upper-middle income and represents a vastly different context to the SSA Malawian economic setting that is the focus of our analysis.

In order to improve our understanding of the relationship between economic instability and mental health in LMICs, this paper models the impact of negative economic shocks on the mental health of Malawian adults age 45 years and older from 2012 through 2018. Furthermore, our analysis addresses the methodological limitation in current cross-sectional research exploring economic shocks and mental health. Specifically, exposures to economic shocks and mental health are likely to be determined by time-invariant individual specific factors such as personality disposition and demographic characteristics. We address this issue by utilizing fixed-effects panel data models that control for these unobservables. Lastly, we quantify the effect of experiencing an additional economic shock on the mental health of Malawian adults. We focus on the relationship between negative economic shocks and depressive and anxiety disorders, the most common mental illnesses, which we refer to using the more general term ‘mental health’.

## 2 Materials and methods

The data used in this study come from the Mature Adults Cohort of the Malawi Longitudinal Study of Families and Health (MLSFH-MAC) (Kohler et al., 2020). The MLSFH-MAC is a population-based cohort study of mature adults aged 45+ years, who are overwhelmingly living in rural communities in three districts in Malawi: Balaka in the south, Mchinji in the central region and Rumphi in the north. The MLSFH-MAC was derived from the Malawi Longitudinal Study of Families and Health (MLSFH) in 2012 (Kohler et al., 2015), with currently follow-up waves in 2013, 2017 and 2018. The original MLSFH sample in 1998 was based on a probabilistic population sample, with the sample being augmented by enrolling adolescents, parents and new spouses of respondents in the later rounds of data collections (for details, see Kohler et al. (2015)). Comparisons of the 2010 MLSFH study population with the rural samples of the Malawi Demographic and Health Survey (DHS) and the Integrated Household Survey 3 (IHS3) reveal that the cohort closely matches the rural sub-sample in the 2010 nationally representative Integrated Household Survey 3 (IHS3) in key observable characteristics (Kohler et al., 2015, 2020). Detailed information on the MLSFH-MAC sampling procedures, comparisons of the study population with nationally representative samples, study design and study instruments are provided in the MLSFH- MAC Cohort Profile (Kohler et al., 2020). While not nationally representative, the MLSFH- MAC broadly represents older persons above age 45 living in rural Malawi where the majority (85%) of all Malawians live. Most of individuals living in these rural areas rely on subsistence farming and engage in manual, intensive physical labor such as home production of crops, complemented by some market activities.

Since it's inception, MLSFH-MAC collects extensive information on mental health and specifically depression and anxiety and provides detailed descriptions of individual's exposure to economic shocks. Our analysis is longitudinal and includes respondents who participated in the 2012, 2013, 2017 and 2018 waves of the MLSFH-MAC.

We employed two measures of mental health outcomes in this study. Our first dependent variable, the PHQ-9 depression score, ranges from 0 to 27 (from having no depressive symptoms at all to worse depression). The PHQ-9 includes nine questions that ask the respondent to categorize if or how often they have been bothered in the past two weeks by indicators such as: *(1) little interest or pleasure in doing things; (2) feeling down, depressed, or hopeless ; … (6) feeling bad about yourself–or that you are a failure or have let yourself or your family down; … (9) thoughts that you would be better off dead or of hurting yourself in some way.* Response categories for all questions in the PHQ-9 range from 0 (not at all) to 3 (nearly every day). We computed an overall depression score, PHQ-9 score, equal to the sum of scores from the PHQ-9 instrument. Based on the PHQ-9 guidelines, the clinical significance of depression is classified as follows: 0–4 indicates none/minimal depression; 5–9 indicates mild depression; 10–14 indicates moderate depression; 15–19 indicates moderately severe depression; and 20–27 indicates severe depression (Kroenke et al., 2010).

The second dependent variable, the GAD-7 anxiety score, ranges from 0 to 21 (from least to worst anxiety). GAD-7 includes 7 questions that ask the respondent to categorize if or how often they have been bothered in the past four weeks by indicators such as the following: *(1) feeling nervous, anxious, or on edge; (2) feeling restless so that it is hard to sit still; ... (6) trouble concentrating on things such as farming, chatting with friends, weaving, or carving?; (7) becoming easily annoyed or irritable?* Similarly, to PHQ-9, response categories for all questions in the GAD-7 range from 0 (not at all) to 3 (nearly every day). An overall anxiety score, the GAD-7 score, was computed as the sum of scores from the GAD-7 instrument. The guidelines for the anxiety measure specify scores of 5, 10, and 15 as cut points for mild, moderate, and severe anxiety (Kroenke et al., 2010).

PHQ-9 and GAD-7 have been shown to be reliable and valid instruments to measure depression and anxiety especially in high-income settings. However, in the low-income context of sub-Saharan Africa, there may be reduced sensitivity and the clinical cut-points for diagnosing major depressive or anxiety disorders remain unclear (Brinkmann et al., 2020; Kessler & Bromet, 2013; Sweetland et al., 2014). Using a qualitative approach and a small sample derived from a larger cohort study, Harrington et al. (2021) found that the PHQ- 9 does not capture all the important symptoms of depression in HIV positive postpartum Malawian women. In contrast, in a PHQ-9 validation study among individuals with type-2 diabetes who attended non-communicable diseases (NCD) clinics in Malawi, Udedi et al. (2019) concluded that the PHQ-9 did generally correctly identify depression. Further, although not equivalent to the PHQ-9 or GAD-7, Ohrnberger et al. (2020a) validated the SF-12 mental and physical health instruments in Malawi suggesting these types of tools capturing differences in mental and physical health among older individuals are applicable in this context.^1^

Our analyses of mental health do not involve qualitatively distinguishing between depressed, mildly depressed, and non-depressed statuses because the respective cut-points for making this distinction in the SSA context remain unclear. Instead, we use a linear specification of the PHQ-9 depression and GAD-7 anxiety scores. These scores do not correspond to a clinical diagnosis of depression or anxiety, but reflect relevant symptomatology. Individuals recording higher scores experience a higher number of depressive or anxiety symptoms. Therefore, we can evaluate a range of depressive and anxiety states without relying on a classification scheme that may not be fully applicable to the low-income country context being studied in this paper.

The central explanatory variable in our study, *Total Negative Economic Shocks*, is a measure of all negative economic shocks experienced in a given year. Respondents were asked if negative events had affected their household in the past two years (2012, 2017, and 2018) or in the past year (2013). The negative shocks included: death or serious illness of an important adult member or someone who provides support for the respondents or the respondent's family; poor crop yields, loss of crops due to disease or pests, or loss of livestock due to theft or disease, or loss of fertilizer coupon; loss of source of income such as loss of employment or business failure; breakup of household such as divorce; and damage to house due to fire, flood, etc. Subsequently, the number of negative economic shocks was added together for each respondent to calculate a total.^2^ Our analyses estimate ordinary least squares panel regressions with fixed effects for individual respondents and year of survey and report robust standard errors clustered on individuals. The estimating equation is

(1) *Y*_*it*_ = *β*_0_ + *β*_1_Total Economic Shocks_*it*_ + *δ***X**_**it**_ + *α*_*i*_ + *γ*_*t*_ + *ε*_*it*_

where *Y*_*it*_ is the outcome (depression or anxiety score) for an individual *i* at time *t*, *Total Economic Shocks*_*it*_ is the number of shocks experienced by *i* in the one to two year period prior to *t*, **X**_**it**_ are other time-varying characteristics of *i* at time time *t*, *α*_*i*_ is an individual fixed effect, *γ*_*t*_ is a wave fixed effect, and *ε*_*it*_ is the residual for individual *i* at time *t*.

A common concern for cross-sectional analyses is that the *α*_*i*_'s are also correlated with *Total Economic Shocks*_*it*_ leading conventional cross-sectional estimates to be biased. However, these individual fixed-effects regressions control for time-invariant unobserved heterogeneity. We conduct a Hausman test to evaluate if the fixed effects specification is preferred to a random effects alternative. It should be clear, though, that time-variant unobservables could still be a source of bias in our panel analyses. To confirm the direction of the fixed effects results are accurate, we also estimate individual level pooled cross-sectional ordinary least squares regressions, regressing PHQ-9 score or GAD-7 score on Total Negative Economic Shocks and the demographic control variables with year fixed effects (see Appendix Tables 4 and 5). We report heteroskedastic robust standard errors clustered on individuals. In our cross-sectional specifications, we control for age group (40–54, 55–64, 65–74, 75+), gender, current marital status (married vs. separated/widowed) and region.^3^

In Table 1 we report the summary statistics for the sample. The sample sizes for 2012, 2013, 2017, and 2018 were 1,296, 1,233, 1,750, and 1,643 respectively. Females on average had higher depression and anxiety scores across all years. Furthermore, females reported experiencing more negative economic shocks. Generally, PHQ-9 scores, GAD-7 scores, and number of negative economic shocks were higher in 2017 and 2018 than in 2012 and 2013.

**Table 1:** Summary Statistics from the MLSFH-MAC Sample Population

	2012		2013		2017		2018	
	Women	Men	Women	Men	Women	Men	Women	Men
Number of Observations	760	536	706	527	1,024	726	976	667

Age Group								
40-54	0.52	0.43	0.52	0.44	0.45	0.38	0.42	0.36
55-64	0.25	0.31	0.25	0.31	0.26	0.29	0.28	0.28
65-74	0.14	0.18	0.15	0.18	0.16	0.19	0.18	0.21
75+	0.08	0.08	0.08	0.08	0.13	0.14	0.13	0.15

Married	0.63	0.95	0.61	0.95	0.59	0.93	0.59	0.94

Region								
Mchinji	0.29	0.32	0.28	0.32	0.30	0.36	0.29	0.37
Balaka	0.39	0.33	0.38	0.33	0.37	0.32	0.37	0.31
Rumphi	0.33	0.35	0.34	0.35	0.33	0.33	0.33	0.33

PHQ-9 Score	3.53	2.53	3.28	2.17	4.67	3.66	4.27	3.00
	(3.86)	(3.58)	(4.01)	(3.34)	(4.41)	(4.04)	(4.25)	(3.69)
GAD-7 Score	2.84	2.16	3.01	2.09	3.97	2.98	3.48	2.46
	(2.62)	(2.43)	(3.24)	(2.81)	(3.59)	(3.20)	(3.34)	(2.89)
Total Economic Shocks	1.39	1.26	1.19	1.03	1.79	1.66	1.55	1.41
	(0.92)	(0.93)	(0.93)	(0.93)	(0.98)	(0.97)	(0.97)	(0.91)

## 3 Results

**Fig. 1:**
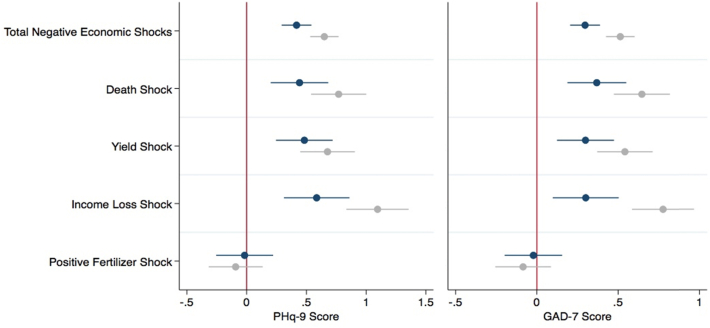
Economic shocks and depression (PHQ-9 score) and anxiety (GAD-7) fixed effect panel estimates and pooled cross-sectional estimates. **Note:** This figure reports OLS fixed effects regression and pooled cross-sectional estimates of PHQ-9 Score and GAD-7 Score on Total Economic Shocks, Death of Family Member Shocks, Income Shocks, Yield Shocks, and Positive Fertilizer Shocks.

Fig. 1 shows the fixed effect panel estimates for PHQ-9 Score and GAD-7 Score (reported in dark color), and the coefficient estimates for the pooled cross-sectional analysis with demographic controls (reported in grey color). Total Negative Economic Shocks, Death of Family Member Shocks, Income Loss Shocks and Yield Shocks all have positive and statistically significant coefficients for each mental health outcome across both specifications.^4^ This indicates a positive relationship between economic insecurity and higher levels of depression and/or anxiety.

Table 2, Table 3 report in further detail the coefficient estimates for the fixed effects analysis that were previously reported in Fig. 1. For the fixed effects analysis, an increase of one shock for Total Negative Economic Shocks is associated with a 0.417 increase in the PHQ-9 score (p < 0.001). Similarly, an increase of one shock for Total Negative Economic Shocks is associated with a 0.296 increase in the GAD-7 score (p *<* 0.001). This relationship was also observed in the pooled cross-sectional analysis with demographic controls. A one-unit increase for Total Negative Economic Shocks, a little greater than one standard deviation, was associated with a 0.651 increase in the PHQ-9 score and 0.513 increase in the GAD-7 score (see Appendix Table 4, Table 5). This positive association is also observed for important components of the Total Economic Shocks index including Death of Family Member Shocks, Yield Shocks, and Income Loss Shocks.

**Table 2:** Economic shocks and depression (PHQ-9 score) fixed effect panel estimates

	(1)	(2)	(3)	(4)	(5)
Total Negative Economic Shocks	0.417∗∗∗				
	(0.064)				
Death of Family Member Shocks		0.442∗∗∗			
		(0.123)			
Yield Shocks			0.483∗∗∗		
			(0.121)		
Income Loss Shocks				0.586∗∗∗	
				(0.140)	
Positive Fertilizer Shocks					−0.0170
					(0.122)

Individual Fixed Effects	Yes	Yes	Yes	Yes	Yes
Year Fixed Effects	Yes	Yes	Yes	Yes	Yes
					
Observations	5333	5349	5350	5346	5343
*R*^2^	0.080	0.072	0.072	0.074	0.068
Hausman Test (p-values)	0.000	0.000	0.000	0.000	0.000

Standard errors in parentheses.

*p < 0*.*05, ∗∗ *p <* 0*.*01, ∗∗∗ *p <* 0*.*001.

**Note:** This table reports OLS fixed effects regression estimates of PHQ-9 Score on Total Economic Shocks, Death of Family Member Shocks, Income Shocks, Yield Shocks, and Positive Fertilizer Shocks. The table reports the coefficient estimates, heteroskedastic robust standard errors clustered on individuals in parentheses, and p-values.

**Table 3:** Economic shocks and anxiety (GAD-7 score) fixed effect panel estimates

	(1)	(2)	(3)	(4)	(5)
Total Negative Economic Shocks	0.296∗∗∗				
	(0.047)				
Death of Family Member Shocks		0.369∗∗∗			
		(0.092)			
Yield Shocks			0.299∗∗∗		
			(0.090)		
Income Loss Shocks				0.301∗∗	
				(0.104)	
Positive Fertilizer Shocks					−0.0215
					(0.090)

Individual Fixed Effects	Yes	Yes	Yes	Yes	Yes
Year Fixed Effects	Yes	Yes	Yes	Yes	Yes
					
Observations	5557	5573	5574	5571	5567
*R*^2^	0.066	0.060	0.059	0.058	0.056
Hausman Test (p-values)	0.000	0.000	0.000	0.000	0.000

Standard errors in parentheses.

*p <* 0*.*05, ∗∗ *p <* 0*.*01, ∗∗∗ *p <* 0*.*001.

**Note:** This table reports OLS fixed effects regression estimates of GAD-7 Score on Total Economic Shocks, Death of Family Member Shocks, Income Shocks, Yield Shocks, and Positive Fertilizer Shocks. The table reports the coefficient estimates, heteroskedastic robust standard errors clustered on individuals in parentheses, and p-values.

To investigate the hypothesis that the relationship between the experience of economic shocks and mental health differs by gender or marital status, in additional pooled cross-sectional analyses we included an interaction term between women and Total Economic Shocks and married and Total Economic Shocks (see Appendix Table 9, Table 10, Table 11, Table 12). Both of these terms were not statistically significant suggesting the effect of negative shocks does not differ for women and men and for those married and unmarried.

In contrast to the cross-sectional estimates, the fixed-effects estimates control for all time constant unobserved characteristics of individuals as well as common shocks across individuals. Although the pooled cross-sectional estimates are useful as a benchmark they generally do not allow for inferring a causal relationship. Under a strong but not unreasonable assumption that the economic shocks are uncorrelated with other changes that might influence mental health outcomes, the fixed effects estimates have a causal interpretation: negative economic shocks induce depression and anxiety and this is also the case in a LIC setting with widespread poverty. Similarly to patterns documented in high-income countries, our results support the conclusion that economically vulnerable populations are at risk for worsening mental health when under further economic stress. This finding is of particular importance since the relationship between negative economic shocks and depression and anxiety have been mostly characterized in high-income countries.

We also investigate whether the association between economic events and mental health is exclusively due to encounter of negative shocks. We examine this by analyzing the relationship between the experience of a positive economic shock—receiving fertilizer subsidy—and the PHQ-9 and GAD-7 scores. Fig. 1 reports the fixed effect estimates and pooled cross-sectional estimates for the impact of positive fertilizer shocks on GAD-7 score and PhQ-9 score. For both GAD-7 and PHQ-9 the point estimates in both model specifications are essentially zero. These analyses thus suggests that only negative economic shocks have a substantial negative effect on the PHQ-9 and GAD-7, while positive economic shocks do not impact mental well-being in this low-income setting.

## 4 Discussion

In the 2019 Global Burden of Disease Study depressive disorders were the thirteenth leading cause of disability-adjusted life-years (DALYs) for all ages (Lancet, 2020). Moreover, since 1990 depressive disorders have gone from only accounting for 1.1 percent of all DALYs to 1.8 percent, or 61.1 percentage change in the number of DALYs 1990–2019. Previous research in high-income countries has shown negative economic events like job loss, income loss, and unemployment have substantial negative consequences for mental health (Alley et al., 2011; Kuhn et al., 2009; Madianos et al., 2011; Frasquilho et al., 2016; Kämpfen et al., 2020; Witteveen & Velthorst, 2020). Additionally, there is some evidence in high-income countries that socioeconomically disadvantaged individuals are especially vulnerable to deteriorating mental health under economic stress (Kämpfen et al., 2020). In SSA positive economic shocks have been established to causally improve mental health via cash-transfer and other anti-poverty experiments (Haushofer & Shapiro, 2016; Kilburn et al., 2016; Angeles et al., 2019; Ohrnberger et al., 2020b,a). Considering the negative impact depression and anxiety can have on individual productivity (Canavan et al., 2013; Kohler et al., 2017; Lund et al., 2018), the high prevalence of depression and anxiety in SSA (Brinkmann et al., 2020; Kohler et al., 2017), and the frequent exposure to negative economic shocks in this context, understanding how negative shocks affect individual's mental health is important and of high relevance for public health policies.

Our analyses find a strong positive relationship between exposure to negative economic events and increased depression and/or anxiety. The fixed effects results and pooled cross-sectional analysis with demographic controls support the conclusion that negative economic shocks lead to higher depression and anxiety in very low-income settings. Death of a family member, loss of agriculture yield, and income loss had particularly strong effects. Within our limited exploration of positive shocks, we found fertilizer subsidies do not hold the same pattern as negative shocks and were not significantly associated with improved depression or anxiety within a fixed effects analysis.

A few limitations to the study should be noted. The PHQ-9 and GAD-7 instruments to measure depression and anxiety were developed in high-income settings, and the validity and sensitivity of these tools in low-income context is less certain. Therefore, it is difficult to be sure of the magnitude of the relationships we found because it is unclear how the PHQ-9 and GAD-7 tools work in resource limited and low-literacy settings. Also, MLSFH-MAC collects information about the shock associated with a death within the family, but does not capture the gender of the family member that has died. Therefore, we are not able to assess possible pathways how this shock affects respondents as there may be different social and economic consequences for a death of an important adult female versus male family member. Lastly, the sample we rely on only surveyed older adults 45+ years old making it impossible to conclude how negative economic events affect the mental well-being of younger potentially more resilient age groups.

Our findings are important for three reasons. First, our analyses suggest that negative economic shocks do have an impact in LIC settings similar to those documented in high-income countries. Despite experiencing shocks to a large extent dissimilar to those in high-income countries, individuals at high levels of poverty still suffer mental health consequences. Second, although some prior studies have assessed the relationship between economic shocks and mental health in low-income settings these analyses mainly relied on cross-sectional data. Cross-sectional analyses may be biased if there are unobserved characteristics of individual or the places they live in that make experiencing negative economic shocks and depression and/or anxiety more likely. In contrast, we employ fixed effects analysis that controls for time invariant unobservables providing more credible estimates of the causal link between economic shocks and worsening mental health. Lastly, this paper contributes to the limited amount of literature covering mental health in SSA. By starting to understand what fac-tors affect mental health in SSA global aide organizations and governments can be better informed on how to approach the increasing burden of depression, anxiety and other mental health problems. Hence, policy makers interested in addressing mental health in low-income countries such as Malawi should consider economic safety nets as a mean to prevent declines in mental health.

## Ethics statement

The data collections of the MLSFH-MAC and MLSFH have been approved by the IRB Board at the University of Pennsylvania (IRB Protocols 815 016 and 826828), and in Malawi, the MLSFH-MAC and MLSFH research has been approved by the Ethics Committee of the College of Medicine, Malawi (COMREC, Protocols P01/12/1165 and P.04/17/2160) and the National Health Sciences Research Committee (NHSRC, Protocol 19/01/2214)

## CRediT author statement

**Ally Scheve**: Conceptualization, formal analysis, writing original draft and revised manuscript; **Chiwoza Bandawe:** helped design the study instruments; **Hans-Peter Kohler:** Conceptualization, design of study instruments, data collection, methodology, writing of original draft and revised manuscript, fund acquisition; **Iliana V. Kohler:** Conceptualization, methodology, study instruments design, data collection, supervision, writing of original draft and revised manuscript, fund acquisition.

## Figures and Tables

**Table 4:** Economic Shocks and Depression (PHQ-9 Score) Pooled Cross-Sectional Estimates

	(1)	(2)	(3)	(4)	(5)
Total Negative Economic Shocks	0.651^∗∗∗^				
	(0.061)				
Death of Family Member Shocks		0.770^∗∗∗^			
		(0.118)			
Income Loss Shocks			1.096^∗∗∗^		
			(0.133)		
Yield Shocks				0.677^∗∗∗^	
				(0.117)	
Positive Fertilizer Shocks					-0.0918
					(0.115)
Women	0.933^∗∗∗^	0.908^∗∗∗^	0.991^∗∗∗^	0.973^∗∗∗^	0.978^∗∗∗^
	(0.134)	(0.137)	(0.135)	(0.136)	(0.137)
Age 55-64	0.336^∗∗^	0.311^∗^	0.304^∗^	0.304^∗^	0.289^∗^
	(0.123)	(0.126)	(0.124)	(0.125)	(0.126)
Age 65-74	1.332^∗∗∗^	1.273^∗∗∗^	1.283^∗∗∗^	1.275^∗∗∗^	1.254^∗∗∗^
	(0.180)	(0.184)	(0.182)	(0.184)	(0.185)
Age 75+	3.245^∗∗∗^	3.068^∗∗∗^	3.167^∗∗∗^	3.136^∗∗∗^	3.061^∗∗∗^
	(0.279)	(0.279)	(0.278)	(0.282)	(0.280)
Married	-0.572^∗∗∗^	-0.661^∗∗∗^	-0.680^∗∗∗^	-0.685^∗∗∗^	-0.671^∗∗∗^
	(0.172)	(0.173)	(0.172)	(0.173)	(0.175)
Balaka	-0.813^∗∗∗^	-0.682^∗∗∗^	-0.793^∗∗∗^	-0.837^∗∗∗^	-0.753^∗∗∗^
	(0.171)	(0.171)	(0.172)	(0.175)	(0.174)
Rumphi	-0.713^∗∗∗^	-0.787^∗∗∗^	-0.792^∗∗∗^	-0.779^∗∗∗^	-0.807^∗∗∗^
	(0.156)	(0.159)	(0.157)	(0.159)	(0.160)

Observations	5329	5345	5342	5346	5339
*R*^2^	0.139	0.125	0.131	0.122	0.117
Fixed Year Effects	Yes	Yes	Yes	Yes	Yes

**Table 5:** Economic Shocks and Anxiety (GAD-7 Score) Pooled Cross-Sectional Estimates

	(1)	(2)	(3)	(4)	(5)
Total Negative Economic Shocks	0.513^∗∗∗^				
	(0.045)				
Death of Family Member Shocks		0.646^∗∗∗^			
		(0.088)			
Income Loss Shocks			0.776^∗∗∗^		
			(0.097)		
Yield Shocks				0.542^∗∗∗^	
				(0.087)	
Positive Fertilizer Shocks					-0.0847
					(0.087)
Women	0.793^∗∗∗^	0.772^∗∗∗^	0.837^∗∗∗^	0.827^∗∗∗^	0.831^∗∗∗^
	(0.102)	(0.103)	(0.103)	(0.103)	(0.104)
Age 55-64	0.372^∗∗∗^	0.350^∗∗∗^	0.342^∗∗∗^	0.345^∗∗∗^	0.330^∗∗^
	(0.100)	(0.101)	(0.100)	(0.101)	(0.101)
Age 65-74	1.107^∗∗∗^	1.058^∗∗∗^	1.064^∗∗∗^	1.057^∗∗∗^	1.044^∗∗∗^
	(0.132)	(0.134)	(0.134)	(0.134)	(0.136)
Age 75+	2.716^∗∗∗^	2.582^∗∗∗^	2.650^∗∗∗^	2.637^∗∗∗^	2.579^∗∗∗^
	(0.216)	(0.215)	(0.214)	(0.218)	(0.216)
Married	-0.339^∗∗^	-0.405^∗∗^	-0.426^∗∗∗^	-0.428^∗∗∗^	-0.418^∗∗^
	(0.127)	(0.128)	(0.128)	(0.128)	(0.129)
Balaka	-0.245^∗^	-0.140	-0.222	-0.274^∗^	-0.205
	(0.124)	(0.125)	(0.125)	(0.127)	(0.127)
Rumphi	-0.195	-0.248^∗^	-0.254^∗^	-0.242^∗^	-0.265^∗^
	(0.120)	(0.122)	(0.122)	(0.123)	(0.124)

Observations	5553	5569	5567	5570	5563
*R*^2^	0.137	0.124	0.126	0.120	0.115
Fixed Year Effects	Yes	Yes	Yes	Yes	Yes

**Table 6:** Economic Shocks and Depression (PHQ-9 Score) Fixed Effect Panel Estimates (Only shocks within one year of survey)

	(1)	(2)	(3)	(4)	(5)
Total Negative Economic Shocks	0.369∗∗∗				
	(0.062)				
Death of Family Member Shocks		0.317∗			
		(0.132)			
Yield Shocks			0.408∗∗∗		
			(0.107)		
Income Loss Shocks				0.534∗∗∗	
				(0.144)	
Positive Fertilizer Shocks					0.0641
					(0.120)
Individual Fixed Effects	Yes	Yes	Yes	Yes	Yes
Year Fixed Effects	Yes	Yes	Yes	Yes	Yes
					
Observations	5334	5349	5350	5346	5343
*R*^2^	0.078	0.070	0.072	0.073	0.068
Hausman Test (p-values)	0.000	0.000	0.000	0.000	0.000

Standard errors in parentheses.

*p < 0*.*05.

∗∗ *p <* 0*.*01.

∗∗∗ *p <* 0*.*001.

**Note:** This table reports OLS fixed effects regression estimates of PHQ-9 Score on a second calculation of Total Economic Shocks, Death of Family Member Shocks, Income Shocks, Yield Shocks, and Positive Fertilizer Shocks which only includes shocks within one year of the survey administration year. The table reports the coefficient estimates, heteroskedastic robust standard errors clustered on individuals in parentheses.

**Table 7:** Economic Shocks and Anxiety (GAD-7 Score) Fixed Effect Panel Estimates (Only shocks within one year of survey)

	(1)	(2)	(3)	(4)	(5)
Total Negative Economic Shocks	0.233∗∗∗				
	(0.048)				
Death of Family Member Shocks		0.263∗∗			
		(0.100)			
Yield Shocks			0.228∗∗		
			(0.083)		
Income Loss Shocks				0.267∗	
				(0.106)	
Positive Fertilizer Shocks					0.0848
					(0.091)
Individual Fixed Effects	Yes	Yes	Yes	Yes	Yes
Year Fixed Effects	Yes	Yes	Yes	Yes	Yes
					
Observations	5558	5573	5574	5571	5567
*R*^2^	0.063	0.058	0.058	0.058	0.056
Hausman Test (p-values)	0.000	0.000	0.000	0.000	0.000

Standard errors in parentheses.

*p < 0*.*05, ∗∗ *p <* 0*.*01, ∗∗∗ *p <* 0*.*001.

**Note:** This table reports OLS fixed effects regression estimates of GAD-7 Score on a second calculation of Total Economic Shocks, Death of Family Member Shocks, Income Shocks, Yield Shocks, and Positive Fertilizer Shocks which only includes shocks within one year of the survey administration year. The table reports the coefficient estimates, heteroskedastic robust standard errors clustered on individuals in parentheses.

**Table 8:** Correlation Coefficient Between PHQ-9 and GAD-7 Scores and General Mental and Physical Health and Well-Being Measures

	Life Satisfaction	Self Health Rating	Feeling Down	SF-12 Mental Health	SF-12 Physical Health
PHQ-9	−0.317	−0.444	0.498	−0.591	−0.548
GAD-7	−0.319	−0.456	0.478	−0.552	−0.570

**Note:** This table reports correlation coefficients between PHQ-9 and GAD-7 Scores and general mental and physical health and well-being measures. Life satisfaction is measured with the question: How satisfied are you with your life, all things considered? Respondents reported a self-rated score from 1 to 5 where 1 is least satisfied and 5 is most satisfied. Self Health Rating is measured on a 1 to 5 scale where 1 is poor health and 5 is excellent health. Lastly, Feeling Down is measured with the question: How much of the time during the past 4 weeks have you felt downhearted and depressed? Respondents reported a number on a scale of 1–5 where 1 is none of the time and 5 is all of the time. The SF-12 Mental Health and Physical Health Scores use a questionnaire to assess general well-being with higher scores being associated with better overall health.

**Table 9:** Total Economic Shocks and Depression (PHQ-9) with Interaction Terms Fixed Effects Analysis

	(1)	(2)
Total Negative Economic Shocks	0.458∗∗∗	0.461∗∗∗
	(0.095)	(0.108)
Female*Total Negative Economic Shocks	−0.0677	
	(0.123)	
Married*Total Negative Economic Shocks		−0.0626
		(0.113)
Observations	5333	5333
*R*^2^	0.080	0.080
Individual Fixed Effects	Yes	Yes
Year Fixed Effects	Yes	Yes
Hausman Test (p-values)	0.000	0.000

Standard errors in parentheses.

*p < 0*.*05.

∗∗ *p <* 0*.*01.

∗∗∗ *p <* 0*.*001.

**Note:** This table reports OLS fixed effects regression estimates of PHQ-9 Score on Total Economic Shocks with interaction terms for gender and marriage status. The table reports the coefficient estimates, heteroskedastic robust standard errors clustered on individuals in parentheses.

**Table 10:** Total Economic Shocks and Anxiety (GAD-7) with Interaction Terms Fixed Effects Analysis

	(1)	(2)
Total Negative Economic Shocks	0.289^∗∗∗^	0.263^∗∗^
	(0.071)	(0.081)
Female*Total Negative Economic Shocks	0.0124	
	(0.093)	
Married*Total Negative Economic Shocks		0.0490
		(0.085)
Observations	5557	5557
*R*^2^	0.066	0.067
Individual Fixed Effects	Yes	Yes
Year Fixed Effects	Yes	Yes
Hausman Test (p-values)	0.000	0.000

Standard errors in parentheses.

*p < 0*.*05.

∗∗ *p <* 0*.*01.

∗∗∗ *p <* 0*.*001.

**Note:** This table reports OLS fixed effects regression estimates of GAD-7 Score on Total Economic Shocks with interaction terms for gender and marriage status. The table reports the coefficient estimates, heteroskedastic robust standard errors clustered on individuals in parentheses.

**Table 11:** Total Economic Shocks and Depression (PHQ-9) with Interaction Terms Pooled Cross-Sectional Estimates

	(1)	(2)
Total Negative Economic Shocks	0.734^∗∗∗^	0.449^∗∗∗^
	(0.090)	(0.130)
Female*Total Negative Economic Shocks	-0.141	
	(0.117)	
Married*Total Negative Economic Shocks		0.281
		(0.143)
Female	1.129^∗∗∗^	0.926^∗∗∗^
	(0.190)	(0.134)
Age 55-64	0.333^∗∗^	0.330^∗∗^
	(0.123)	(0.123)
Age 65-74	1.325^∗∗∗^	1.318^∗∗∗^
	(0.181)	(0.181)
Age 75+	3.240^∗∗∗^	3.224^∗∗∗^
	(0.279)	(0.279)
Married	-0.579^∗∗∗^	-1.007^∗∗∗^
	(0.172)	(0.278)
Balaka	-0.813^∗∗∗^	-0.816^∗∗∗^
	(0.171)	(0.170)
Rumphi	-0.712^∗∗∗^	-0.706^∗∗∗^
	(0.156)	(0.156)

Observations	5329	5329
*R*^2^	0.139	0.140
Fixed Year Effects	Yes	Yes

**Table 12:** Total Economic Shocks and Depression (PHQ-9) with Interaction Terms Pooled Cross-Sectional Estimates

	(1)	(2)
Total Negative Economic Shocks	0.519^∗∗∗^	0.380^∗∗∗^
	(0.0657)	(0.0955)
Female*Total Negative Economic Shocks	-0.00866	
	(0.0879)	
Married*Total Negative Economic Shocks		0.185
		(0.106)
Female	0.805^∗∗∗^	0.789^∗∗∗^
	(0.145)	(0.101)
Age 55-64	0.372^∗∗∗^	0.368^∗∗∗^
	(0.100)	(0.100)
Age 65-74	1.106^∗∗∗^	1.098^∗∗∗^
	(0.132)	(0.132)
Age 75+	2.715^∗∗∗^	2.702^∗∗∗^
	(0.216)	(0.217)
Married	-0.339^∗∗^	-0.625^∗∗^
	(0.127)	(0.203)
Balaka	-0.245^∗^	-0.247^∗^
	(0.124)	(0.124)
Rumphi	-0.195	-0.190
	(0.120)	(0.121)
		
Observations	5553	5553
*R*^2^	0.137	0.138
Fixed Year Effects	Yes	Yes

## Footnotes

1 See Appendix Table 8 for correlation coefficients between PHQ-9 and GAD-7 scores and general well-being measures.

2 A second version of the “Total Economic Shocks”, “Death of Family Member Shocks”, “Income Loss Shocks”, “Yield Shocks”, and “Positive Fertilizer Shocks” variables was also calculated. The second versions of these variables only counted shocks that occurred within one year of the survey rather than two. These OLS fixed effects regression results are in Table 6, Table 7 in the appendix yielding similar results.

3 Age misreporting among older individuals is a common problem in a context such as Malawi, where birth registration and birth certificates did not exist in the past. There were 205 respondents in our sample who reported inconsistent age across survey waves and that for at least one year were in the 40–44 age range. For that reason, we included these respondents in the age group 40–54.

4 We conducted a Hausman test to evaluate whether a fixed effects or random effects specification was preferred for the results reported in Fig. 1. For each, we could reject the null hypothesis that coefficients in the fixed effects and random effects models were equal, which suggests that the fixed effects models are preferred.
