# Supplementary material for: Mental health and life-course shocks in a low-income country: Evidence from Malawi
Source: SSM Popul Health. 2022 Apr 26;19:101098. doi: 10.1016/j.ssmph.2022.101098 (PMC9194642; doi:10.1016/j.ssmph.2022.101098)
Supplement: Multimedia component 1 [file mmc1.pdf]

Appendix

Table 4: Economic Shocks and Depression (PHQ-9 Score) Pooled Cross-Sectional Estimates

	(1)	(2)	(3)	(4)	(5)
Total Negative Economic Shocks	0.651***				
	(0.0608)				
Death of Family Member Shocks		0.770***			
		(0.118)			
Income Loss Shocks			1.096***		
			(0.133)		
Yield Shocks				0.677***	
				(0.117)	
Positive Fertilizer Shocks					-0.0918
					(0.115)
Women	0.933***	0.908***	0.991***	0.973***	0.978***
	(0.134)	(0.137)	(0.135)	(0.136)	(0.137)
Age 55-64	0.336**	0.311*	0.304*	0.304*	0.289*
	(0.123)	(0.126)	(0.124)	(0.125)	(0.126)
Age 65-74	1.332***	1.273***	1.283***	1.275***	1.254***
	(0.180)	(0.184)	(0.182)	(0.184)	(0.185)
Age 75+	3.245***	3.068***	3.167***	3.136***	3.061***
	(0.279)	(0.279)	(0.278)	(0.282)	(0.280)
Married	-0.572***	-0.661***	-0.680***	-0.685***	-0.671***
	(0.172)	(0.173)	(0.172)	(0.173)	(0.175)
Balaka	-0.813***	-0.682***	-0.793***	-0.837***	-0.753***
	(0.171)	(0.171)	(0.172)	(0.175)	(0.174)
Rumphi	-0.713***	-0.787***	-0.792***	-0.779***	-0.807***
	(0.156)	(0.159)	(0.157)	(0.159)	(0.160)
Observations	5329	5345	5342	5346	5339
R^2	0.139	0.125	0.131	0.122	0.117
Fixed Year Effects	Yes	Yes	Yes	Yes	Yes

Standard errors in parentheses

* $p < 0.05$, ** $p < 0.01$, *** $p < 0.001$

Note: This table reports OLS pooled cross-sectional regression estimates of PHQ-9 Score on Total Economic Shocks, Death of Family Member Shocks, Income Shocks, Yield Shocks, and Positive Fertilizer Shocks with demographic control variables. The table reports the coefficient estimates and heteroskedastic robust standard errors clustered on individuals in parentheses.

Table 5: Economic Shocks and Anxiety (GAD-7 Score) Pooled Cross-Sectional Estimates

	(1)	(2)	(3)	(4)	(5)
Total Negative Economic Shocks	0.513***				
	(0.0448)				
Death of Family Member Shocks		0.646***			
		(0.0878)			
Income Loss Shocks			0.776***		
			(0.0972)		
Yield Shocks				0.542***	
				(0.0869)	
Positive Fertilizer Shocks					-0.0847
					(0.0874)
Women	0.793***	0.772***	0.837***	0.827***	0.831***
	(0.102)	(0.103)	(0.103)	(0.103)	(0.104)
Age 55-64	0.372***	0.350***	0.342***	0.345***	0.330**
	(0.0989)	(0.101)	(0.0997)	(0.101)	(0.101)
Age 65-74	1.107***	1.058***	1.064***	1.057***	1.044***
	(0.132)	(0.134)	(0.134)	(0.134)	(0.136)
Age 75+	2.716***	2.582***	2.650***	2.637***	2.579***
	(0.216)	(0.215)	(0.214)	(0.218)	(0.216)
Married	-0.339**	-0.405**	-0.426***	-0.428***	-0.418**
	(0.127)	(0.128)	(0.128)	(0.128)	(0.129)
Balaka	-0.245*	-0.140	-0.222	-0.274*	-0.205
	(0.124)	(0.125)	(0.125)	(0.127)	(0.127)
Rumphi	-0.195	-0.248*	-0.254*	-0.242*	-0.265*
	(0.120)	(0.122)	(0.122)	(0.123)	(0.124)
Observations	5553	5569	5567	5570	5563
R^2	0.137	0.124	0.126	0.120	0.115
Fixed Year Effects	Yes	Yes	Yes	Yes	Yes

Standard errors in parentheses

* $p < 0.05$, ** $p < 0.01$, *** $p < 0.001$

Note: This table reports OLS pooled cross-sectional regression estimates of GAD-7 Score on Total Economic Shocks, Death of Family Member Shocks, Income Shocks, Yield Shocks, and Positive Fertilizer Shocks with demographic control variables. The table reports the coefficient estimates and heteroskedastic robust standard errors clustered on individuals in parentheses.

Table 6: Economic Shocks and Depression (PHQ-9 Score) Fixed Effect Panel Estimates (Only shocks within one year of survey)

	(1)	(2)	(3)	(4)	(5)
Total Negative Economic Shocks	0.369***				
	(0.0621)				
Death of Family Member Shocks		0.317*			
		(0.132)			
Yield Shocks			0.408***		
			(0.107)		
Income Loss Shocks				0.534***	
				(0.144)	
Positive Fertilizer Shocks					0.0641
					(0.120)
Individual Fixed Effects	Yes	Yes	Yes	Yes	Yes
Year Fixed Effects	Yes	Yes	Yes	Yes	Yes
Observations	5334	5349	5350	5346	5343
R^2	0.078	0.070	0.072	0.073	0.068
Hausman Test (p-values)	0.000	0.000	0.000	0.000	0.000

Standard errors in parentheses

* $p < 0.05$, ** $p < 0.01$, *** $p < 0.001$

Note: This table reports OLS fixed effects regression estimates of PHQ-9 Score on a second calculation of Total Economic Shocks, Death of Family Member Shocks, Income Shocks, Yield Shocks, and Positive Fertilizer Shocks which only includes shocks within one year of the survey administration year. The table reports the coefficient estimates, heteroskedastic robust standard errors clustered on individuals in parentheses.

Table 7: Economic Shocks and Anxiety (GAD-7 Score) Fixed Effect Panel Estimates (Only shocks within one year of survey)

	(1)	(2)	(3)	(4)	(5)
Total Negative Economic Shocks	0.233***				
	(0.0484)				
Death of Family Member Shocks		0.263**			
		(0.100)			
Yield Shocks			0.228**		
			(0.0831)		
Income Loss Shocks				0.267*	
				(0.106)	
Positive Fertilizer Shocks					0.0848
					(0.0906)
Individual Fixed Effects	Yes	Yes	Yes	Yes	Yes
Year Fixed Effects	Yes	Yes	Yes	Yes	Yes
Observations	5558	5573	5574	5571	5567
R^2	0.063	0.058	0.058	0.058	0.056
Hausman Test (p-values)	0.000	0.000	0.000	0.000	0.000

Standard errors in parentheses

* $p < 0.05$, ** $p < 0.01$, *** $p < 0.001$

Note: This table reports OLS fixed effects regression estimates of GAD-7 Score on a second calculation of Total Economic Shocks, Death of Family Member Shocks, Income Shocks, Yield Shocks, and Positive Fertilizer Shocks which only includes shocks within one year of the survey administration year. The table reports the coefficient estimates, heteroskedastic robust standard errors clustered on individuals in parentheses.

Table 8: Correlation Coefficient Between PHQ-9 and GAD-7 Scores and General Mental Health Measures

	Life Satisfaction	Self Health Rating	Feeling Down	SF-12 Mental Health	SF-12 Physical Health
PHQ-9	-0.317	-0.444	0.498	-0.591	-0.548
GAD-7	-0.319	-0.456	0.478	-0.552	-0.570

Note: This table reports correlation coefficients between PHQ-9 and GAD-7 Scores and general mental health measures. Life satisfaction is measured with the question: How satisfied are you with your life, all things considered? Respondents reported a self-rated score from 1 to 5 where 1 is least satisfied and 5 is most satisfied. Self Health Rating is measured on a 1 to 5 scale where 1 is poor health and 5 is excellent health. Lastly, Feeling Down is measured with the question: How much of the time during the past 4 weeks have you felt downhearted and depressed? Respondents reported a number on a scale of 1 to 5 where 1 is none of the time and 5 is all of the time. The SF-12 Mental Health and Physical Health Scores use a questionnaire to assess general well-being with higher scores being associated with better overall health.

Table 9: Total Economic Shocks and Depression (PHQ-9) with Interaction Terms Fixed Effects Analysis

	(1)	(2)
Total Negative Economic Shocks	0.458***	0.461***
	(0.0948)	(0.108)
Female*Total Negative Economic Shocks	-0.0677	
	(0.123)	
Married*Total Negative Economic Shocks		-0.0626
		(0.113)
Observations	5333	5333
R^2	0.080	0.080
Individual Fixed Effects	Yes	Yes
Year Fixed Effects	Yes	Yes
Hausman Test (p-values)	0.000	0.000

Standard errors in parentheses

* $p < 0.05$, ** $p < 0.01$, *** $p < 0.001$

Note: This table reports OLS fixed effects regression estimates of PHQ-9 Score on Total Economic Shocks with interaction terms for gender and marriage status. The table reports the coefficient estimates, heteroskedastic robust standard errors clustered on individuals in parentheses.

Table 10: Total Economic Shocks and Anxiety (GAD-7) with Interaction Terms Fixed Effects Analysis

	(1)	(2)
Total Negative Economic Shocks	0.289***	0.263**
	(0.0712)	(0.0807)
Female*Total Negative Economic Shocks	0.0124	
	(0.0931)	
Married*Total Negative Economic Shocks		0.0490
		(0.0845)
Observations	5557	5557
R^2	0.066	0.067
Individual Fixed Effects	Yes	Yes
Year Fixed Effects	Yes	Yes
Hausman Test (p-values)	0.000	0.000

Standard errors in parentheses

* $p < 0.05$, ** $p < 0.01$, *** $p < 0.001$

Note: This table reports OLS fixed effects regression estimates of GAD-7 Score on Total Economic Shocks with interaction terms for gender and marriage status. The table reports the coefficient estimates, heteroskedastic robust standard errors clustered on individuals in parentheses.

Table 11: Total Economic Shocks and Depression (PHQ-9) with Interaction Terms Pooled Cross-Sectional Estimates

	(1)	(2)
Total Negative Economic Shocks	0.734***	0.449***
	(0.0895)	(0.130)
Female*Total Negative Economic Shocks	-0.141	
	(0.117)	
Married*Total Negative Economic Shocks		0.281
		(0.143)
Female	1.129***	0.926***
	(0.190)	(0.134)
Age 55-64	0.333**	0.330**
	(0.123)	(0.123)
Age 65-74	1.325***	1.318***
	(0.181)	(0.181)
Age 75+	3.240***	3.224***
	(0.279)	(0.279)
Married	-0.579***	-1.007***
	(0.172)	(0.278)
Balaka	-0.813***	-0.816***
	(0.171)	(0.170)
Rumphi	-0.712***	-0.706***
	(0.156)	(0.156)
Observations	5329	5329
R^2	0.139	0.140
Fixed Year Effects	Yes	Yes

Standard errors in parentheses

* $p < 0.05$, ** $p < 0.01$, *** $p < 0.001$

Note: This table reports OLS pooled cross-sectional regression estimates of PHQ-9 Score on Total Economic Shocks with interaction terms for gender and marriage status and demographic control variables. The table reports the coefficient estimates and heteroskedastic robust standard errors clustered on individuals in parentheses.

Table 12: Total Economic Shocks and Anxiety (GAD-7) with Interaction Terms Pooled Cross-Sectional Estimates

	(1)	(2)
Total Negative Economic Shocks	0.519***	0.380***
	(0.0657)	(0.0955)
Female*Total Negative Economic Shocks	-0.00866	
	(0.0879)	
Married*Total Negative Economic Shocks		0.185
		(0.106)
Female	0.805***	0.789***
	(0.145)	(0.101)
Age 55-64	0.372***	0.368***
	(0.0989)	(0.0991)
Age 65-74	1.106***	1.098***
	(0.132)	(0.132)
Age 75+	2.715***	2.702***
	(0.216)	(0.217)
Married	-0.339**	-0.625**
	(0.127)	(0.203)
Balaka	-0.245*	-0.247*
	(0.124)	(0.124)
Rumphi	-0.195	-0.190
	(0.120)	(0.121)
Observations	5553	5553
R^2	0.137	0.138
Fixed Year Effects	Yes	Yes

Standard errors in parentheses

* $p < 0.05$, ** $p < 0.01$, *** $p < 0.001$

Note: This table reports OLS pooled cross-sectional regression estimates of GAD-7 Score on Total Economic Shocks with interaction terms for gender and marriage status and demographic control variables. The table reports the coefficient estimates and heteroskedastic robust standard errors clustered on individuals in parentheses.
